# Testicular Necrosis Secondary to Incarcerated Inguinal Hernia in Male Infants. Own Observations

**DOI:** 10.34763/devperiodmed.20182201.6570

**Published:** 2018-04-12

**Authors:** Teresa Dudek-Warchoł, Wojciech Gług, Aleksandra Kurek, Przemysław Bombiński, Stanisław Warchoł

**Affiliations:** 1 Department of Pediatric Surgery and Urology, Medical University of Warsaw, Warsaw, Poland; 2 Student’s Society for Pediatric Surgery and Urology, Medical University of Warsaw, Warsaw, Poland; 3 Department of Pediatric Radiology, Medical University of Warsaw, Warsaw, Poland

**Keywords:** hernia incarceration, testicular necrosis, inguinal hernia, uwięźnięcie przepukliny, martwica jądra, przepuklina pachwinowa

## Abstract

One of the possible consequences of incarcerated inguinal hernia in boys is testicular ischemia because of the prolonged compression of spermatic cord structures by the sac contents, resulting in ipsilateral testicular atrophy. This complication is well described in the literature and occurs in 5-34% of patients. The incidence of testicular atrophy secondary to incarcerated hernia is estimated to be 2-3%. Testicular necrosis as the result of hernia incarceration is, however, an extremely rare clinical setting. We present 4 male infants aged 3-10 weeks with inguinal hernia incarceration which led to ipsilateral testicular loss. All the boys had to be operated on because of irreducible incarcerated hernia and in all the cases testicular necrosis was found intraoperatively. The time of incarceration before surgical intervention ranged from 4 to 12 hours (mean 6.75). Our data show that every case of hernia incarceration in a very young male infant requires rapid diagnosis and proper intervention, i.e. surgical treatment, instead of repeated attempts of manual reduction. Ultrasound examination should estimate not only blood flow through the incarcerated intestinal loop, but also through the ipsilateral testis. Moreover, during the operation of the incarcerated hernia in a boy it is necessary to estimate the ipsilateral testis.

## Streszczenie

Jednym z możliwych następstw uwięźnięcia przepukliny pachwinowej u chłopców jest niedokrwienie jądra wskutek przedłużonego ucisku na struktury powrózka nasiennego przez zawartość worka przepuklinowego, z następowym zanikiem jądra. Powyższe powikłanie jest opisywane w piśmiennictwie i występuje u 5-34% pacjentów. Częstość występowania atrofii jądra wtórnej do uwięźnięcia przepukliny pachwinowej oceniana jest na 2% do 3%. Martwica jądra w wyniku uwięźnięcia przepukliny należy do niezmiernie rzadkich zdarzeń. W pracy przedstawiono 4 przypadki chłopców w wieku 3-10 tygodni z uwięźnięciem przepukliny pachwinowej prowadzącym do martwicy jądra. Wszyscy chłopcy byli leczeni operacyjnie z powodu niedającej się odprowadzić manulanie uwięźniętej przepukliny i u wszystkich śródoperacyjnie stwierdzono martwicę jądra po stronie uwięźnięcia. Czas trwania uwięźnięcia do momentu interwencji operacyjnej wynosił od 4 do 12 godzin (średni 6.75). Nasza obserwacja wskazuje na konieczność szybkiego rozpoznania i wdrożenia właściwego postępowania (leczenie operacyjne bez powtarzania kolejnych prób manualnego odprowadzenia przepukliny) w każdym przypadku uwięźnięcia przepukliny u chłopca w wieku wczesoniemowlęcym. Ultrasonograficznie powinien być oceniany nie tylko przepływ przez uwięźniętą pętlę jelitową, ale również przez jądro po stronie uwięźnięcia oraz w czasie operacji uwięźniętej przepukliny konieczna jest ocena jądra po stronie operowanej.

## Introduction

Inguinal hernia in children is one of the most common congenital disorders managed by pediatricians and pediatric surgeons. Inguinal hernia repair is also one of the most common operations performed by pediatric surgeons. The main etiological factor for hernia development in children is prolonged patency of processus vaginalis, therefore indirect inguinal hernia occurs almost exclusively (in about 99% of all cases). In the general pediatric population the overall incidence of inguinal hernia is estimated at approximately 3% to 5%.

Because processus vaginalis plays an important role in testicular descent, male children with hernias outnumber females by an 8:1 to 10:1 ratio [1, 2, 3, 4].

The most serious complication of inguinal hernia is its incarceration. The risk for hernia incarceration is the highest in children less than 1 year old (70% of cases) and decreases with age. Younger age and as well as prematurity are attributed as a risk factor for incarceration [1, 2, 3, 4].

One of the possible consequences of incarcerated inguinal hernia in boys is testicular ischemia as a result of prolonged spermatic cord structure compression [1, 5, 6, 7, 8].

The aim of this study is to present a case series of male infants with testicular necrosis and its loss as a complication of incarcerated inguinal hernia.

## Material and methods

Over the last year 4 boys aged 3, 4, 5 and 10 weeks were diagnosed with irreducible incarcerated inguinal hernia and referred for urgent surgery. After the diagnosis of hernia incarceration at least one attempt to reduce the hernia manually was made. The clinical data of the patients are presented in Table I.

**Table I:** Clinical data of 4 boys with testicular necrosis due to inguinal hernia incarceration. Tabela I. Dane kliniczne 4 chłopców z martwicą jądra wskutek uwięźnięcia przepukliny pachwinowej.

Pt no *Pacjent no*	Age (weeks) *Wiek (tygodnie)*	Side *Strona*	Risk factors *Czynniki ryzyka*	US results *Wynik badania USG*	Time of incarceration *Czas trwania uwięźnięcia*
1	3	left *lewa*	prematurity *wcześniactwo*	incarcerated intestinal loop with grossly diminished blood flow *uwięźnięta pętla jelitowa ze znacznie zmniejszonym przepływem* slightly enlarged ipsilateral testis, abnormal testicular structure with visible blood flow *nieznacznie powiększone jądro, nieprawidłowa struktura jądra, widoczny przepływ krwi przez jądro*	****** 12 hrs *12 godz*.
2	4	right *prawa*		incarcerated intestinal loop with diminished blood flow *uwięźnięta pętla jelitowa ze zmniejszonym przepływem* enlarged ipsilateral testis, normal testicular structure, blood flow not estimated *powiększone jądro, prawidłowa struktura jądra, bez oceny przepływu krwi*	5 hrs *5 godz*.
3	5	right *prawa*		incarcerated intestinal loop with visible blood flow *uwięźnięta pętla jelitowa z widocznym przepływem* enlarged ipsilateral testis, slightly abnormal testicular structure, blood flow not estimated *powiększone jądro, nieco nieprawidłowa struktura jądra, bez oceny przepływu krwi*	6 hrs *6 godz*.
4	10	left *lewa*	prematurity *wcześniactwo*	incarcerated intestinal loop with visible blood flow *uwięźnięta pętla jelitowa z widocznym przepływem* normal ipsilateral testis, blood flow not estimated *prawidłowe jądro, bez oceny przepływu krwi*	4 hrs *4 godz*.

**Fig. 1:**
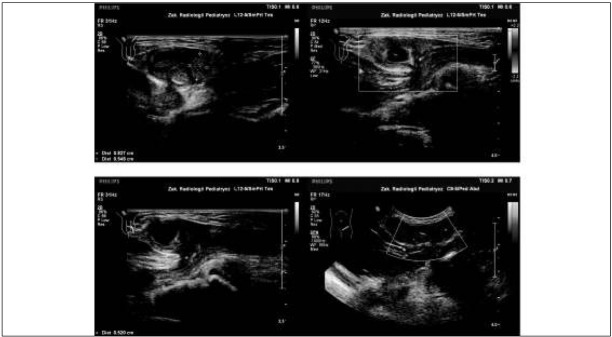
US: incarcerated left inguinal hernia: intestinal loop with grossly diminished blood flow; enlarged ipsilateral testis, abnormal testicular structure, visible blood flow through the testis. Ryc. 1. USG: uwięźnięta lewostronna przepuklina pachwinowa: pętla jelitowa ze znacznie zmniejszonym przepływem; nieznacznie powiększone lewe jądro, nieprawidłowa struktura jądra, widoczny przepływ krwi przez jądro.

In all the boys ultrasound (US) was performed after the recognition of hernia incarceration, although in every case blood flow was checked only within the incarcerated bowel loop. The US revealed the presence of an incarcerated bowel loop within the inguinal canal. In 2 boys enlargement of the ipsilateral testis was diagnosed: in one there was slightly abnormal testicular echogenicity, in the other normal testicular structure. In the third case slight enlargement of the testis was found, with an abnormal structure, but there was visible blood flow, while in the last one the testis was described as completely normal. Only in one out of the 4 cases was there estimated blood flow through the testis (Figure 1).

## Results

**Fig. 2:**
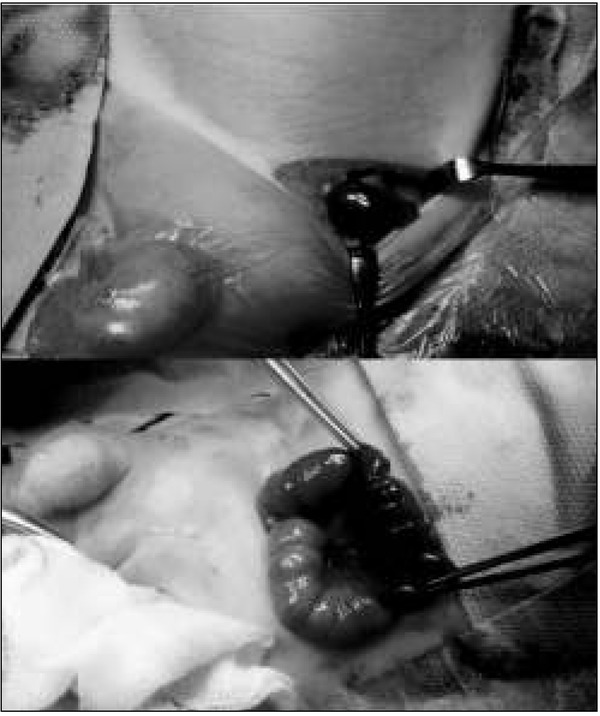
Intraoperative view: incarcerated left inguinal hernia: necrosis of the testis. Ryc. 2. Zdjęcie śródoperacyjne: uwięźnięta lewostronna przepuklina pachwinowa: martwiczo zmienione jądro.

**Fig. 3:**
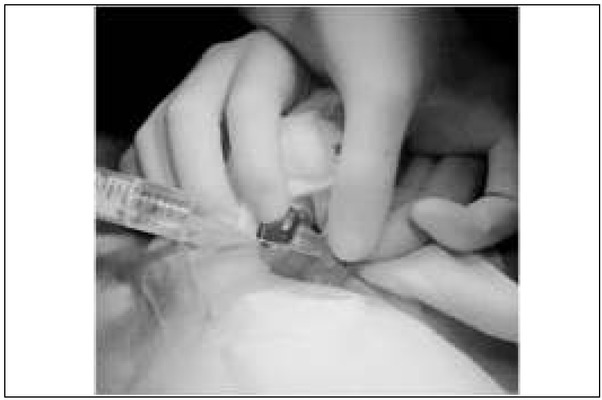
Intraoperative view: local lidocaine injection. Ryc. 3. Zdjęcie śródoperacyjne: miejscowe ostrzyknięcie lignokainą.

Table II presents the details of operative findings, as well as details of applied surgical management. Intraoperatively testicular necrosis with no torsion of spermatic cord structures was found in all 4 boys, despite an incarcerated bowel loop (Figure 2). In 3 the incarcerated intestinal loop estimated as viable was reduced, in the remaining 4^th^ patient bowel resection with primary anastomosis was necessary, because of concomitant bowel necrosis. As the attempts of revascularization of the testes by their warming and local lidocaine injection were ineffective (Figure 3), orchiectomy was performed in all the patients. The postoperative course was uneventful and the patients were discharged home 3 to 6 days after surgery.

**Table II:** Operative findings and details of applied surgical management. Tabela II. Dane śródoperacyjne oraz sposób postępowania.

Pt no *Pacjent no*	Time of incarceration *Czas trwania uwięźnięcia*	Intraoperative findings *Dane śródoperacyjne*	Intervention *Postępowanie*
1	12 hrs *12 godz*.	necrotic incarcerated intestinal loop *martwiczo zmieniona uwięźnięta pętla jelitowa* necrotic ipsilateral testis (no torsion of spermatic cord structures) *martwiczo zmienione jądro (bez skrętu elementów powrózka)*	intestinal resection/anastomosis *resekcja/zespolenie jelita* orchiectomy *usunięcie jądra*
2	5 hrs *5 godz*.	viable incarcerated intestinal loop *przekrwiona, obrzęknięta uwięźnięta pętla jelitowa* necrotic ipsilateral testis (no torsion of spermatic cord structures) *martwiczo zmienione jądro (bez skrętu elementów powrózka)*	hernia reduction *odprowadzenie pętli jelitowej* orchiectomy *usunięcie jądra*
3	6 hrs *6 godz*.	viable incarcerated intestinal loop *przekrwiona, obrzęknięta uwięźnięta pętla jelitowa* necrotic ipsilateral testis (no torsion of spermatic cord structures) *martwiczo zmienione jądro (bez skrętu elementów powrózka)*	hernia reduction *odprowadzenie pętli jelitowej* orchiectomy *usunięcie jądra*
4	4 hrs *4 godz*.	viable incarcerated intestinal loop *przekrwiona, obrzęknięta uwięźnięta pętla jelitowa* necrotic ipsilateral testis (no torsion of spermatic cord structures) *martwiczo zmienione jądro (bez skrętu elementów powrózka)*	hernia reduction *odprowadzenie pętli jelitowej* orchiectomy *usunięcie jądra*

The histopatological examination of the removed testes showed hemorrhagic infarction with necrosis in all the cases.

## Discussion

The vascular supply to the testis may be compromised by the incarcerated bowel, resulting in ischemia of the ipsilateral testis. The possibility of such testicular injury during inguinal hernia incarceration is a known clinical entity with reported occurrence of testicular ischemia from 5 to 34% [1, 5, 6, 7, 8, 9]. Prolonged testicular ischemia can finally result in testicular atrophy. The reported incidence of this complication is 2 to 3% [1, 2, 5, 6, 7, 8].

Within an incarcerated hernia which undergoes operative treatment, the testis of the affected side is usually found to be edematous and also somewhat cyanotic. Generally, unless the gonad is frankly necrotic, it is postulated to preserve the testis. In such cases the parents of each boy should be informed about the possibility of testicular diminishing and atrophy in the future [1, 2, 5, 6].

A very limited number of reports regarding testicular infarction/necrosis/gangrene caused by an incarcerated inguinal hernia are available in the literature and almost all of them are case reports. So far one can find 16 reports in Pub Med [5, 6, 7, 8, 10, 11, 12, 13, 14, 15, 16, 17, 18, 19, 20, 21, 22].

In most of the published reports it is stated that testicular injury was not anticipated but was incidentally found during the operation. All the cases described referred to boys who required emergency operations because of an irreducible hernia. Moreover, testicular injury was typically noted in very young male infants, usually less than 3 months of age. All of our patients were also within the first 3 months of their lives (the oldest one was 10 weeks) and all were referred for urgent surgery because of irreducible incarcerated inguinal hernias.

To date we have not found a similar case series presented in the literature. We were able to find only two similar single cases, including one with necrosis of an undescended testis by an incarcerated hernia [21, 22].

## Conclusions

Our observations confirm that the reduction of the incarcerated inguinal hernia should be performed as early as possible, not only to preserve the bowel, but also to avoid excess pressure on the spermatic cord structures with possible testicular injury. On the other hand it seems that incarceration in a very young male infant requires proper intervention, i.e. operative treatment instead of repeated attempts of manual reduction, so as to avoid additional injury to the testis. It is also worth emphasizing that the initial ultrasound examination should estimate not only blood flow through the incarcerated inguinal loop, but also through the ipsilateral testis. During the operation of an incarcerated hernia in a boy it is necessary to estimate the ipsilateral testis.
